# The influence of resection margin width in patients with intrahepatic cholangiocarcinoma: a meta-analysis

**DOI:** 10.1186/s12957-023-02901-5

**Published:** 2023-01-20

**Authors:** Yu-Shi Dai, Hai-Jie Hu, Tian-run Lv, Ya-Fei Hu, Rui-Qi Zou, Fu-Yu Li

**Affiliations:** Department of Biliary Surgery, West China Hospital of Sichuan University, Chengdu, 610041 Sichuan Province China

**Keywords:** Intrahepatic cholangiocarcinoma, Resection, Survival, Prognosis

## Abstract

**Background:**

Some studies have pointed out that a wide resection margin can improve the prognosis of intrahepatic cholangiocarcinoma, but some researchers disagree and believe that a wide margin may increase complications. The optimal margin length of intrahepatic cholangiocarcinoma is controversial.

**Method:**

The literature was searched in PubMed, MedLine, Embase, the Cochrane Library, and Web of Science until December 31, 2021, to evaluate the postoperative outcomes of patients with different margin width after resection. Odds ratios (ORs) with 95% confidence intervals were used to determine the effect size.

**Result:**

A total of 11 articles were included in this meta-analysis, including 3007 patients. The narrow group had significantly lower 1-, 3-, and 5-year overall survival rates and recurrence-free survival rates than the wide group. Postoperative morbidity and prognostic factors were also evaluated.

**Conclusion:**

A resection margin width of over 10 mm is recommended in intrahepatic cholangiocarcinoma patients, especially in patients with negative lymph node and early tumor stage. When the resection margin width cannot be greater than 10 mm, we should ensure that the resection margin width is greater than 5 mm.

**Supplementary Information:**

The online version contains supplementary material available at 10.1186/s12957-023-02901-5.

## Introduction

Intrahepatic cholangiocarcinoma (ICC) is the second most common primary malignancy in the liver, developing from a secondary bile duct branch [1]. ICC can be classified into three categories based on morphology: mass-forming (MF), intraductal growth (IG), and periductal infiltrative (PI). In recent years, the incidence rate of ICC has risen, particularly in Asia [2–4]. The only cure is curative resection. Unfortunately, most patients are diagnosed at an advanced stage, and only 10% to 20% of them are candidates for surgery, with unresectable candidates having a median survival time (MST) of only 6–9 months [5, 6]. Furthermore, positive margins are thought to be linked to a worse prognosis, and their prevalence ranges from 10 to 40% [7–11]. Even if negative margin (R0) resection is completed, the prognosis is poor, with just a 25–40% 5-year survival rate for these patients [12–14].

The prognosis of ICC is thought to be influenced by tumor size, lymph node status, vascular invasion, pathological categorization, margin width, and other factors [3]. The only one of these criteria that the surgeon can change is the resection margin width. However, it is still debatable whether a greater resection margin is favorable for survival. According to certain experts, patients cannot benefit from a large margin [9, 15]. According to some research, aggressive surgery to achieve a broad margin would increase the risk of complications. In contrast, other studies have found that increasing the margin width significantly improves patient survival [16, 17]. The most appropriate width also cannot be determined. Some studies demonstrate that a 3-mm margin can enhance survival, others show that a 5-mm margin can improve prognosis, while others still believe that a minimum of 10-mm margin width should be assured [9, 18, 19].

Therefore, a more thorough examination is required to arrive at a more precise conclusion on the significance of a broad margin in ICC resection. As a result, a systematic meta-analysis was conducted to assess the benefits of a wider resection margin in ICC.

## Materials and methods

### Study identification and selection

Literature was searched in the databases among Cochrane Library, PubMed, Embase, MedLine, and Web of Science until December 31, 2021. Eligible studies were restricted to comparative studies among different resection margin width groups in patients with ICC. The following keywords were used: ((((((((((intrahepatic cholangiocarcinoma) OR (Cholangiocarcinomas)) OR (Cholangiocellular Carcinoma)) OR (Carcinoma, Cholangiocellular)) OR (Cholangiocellular Carcinomas)) OR (Carcinomas, Cholangiocellular)) OR (Cholangiocarcinoma, Intrahepatic)) OR (Bile Ducts, Intrahepatic)) OR (Bile Duct Neoplasms)) AND ((((resection) OR (excision)) OR (abscission)) OR (hepatectomy))) AND ((((((((margin width) OR (wide margin)) OR (extensive margin)) OR (close margin)) OR (narrow margin)) OR (narrow)) OR (wide)) OR (close)). To extend our research, any relevant meta-analyses and references of the included studies were also screened. This meta-analysis has been registered on PROSPERO with the registration number CRD4202371498 (https://www.crd.york.ac.uk/prospero/display_record.php?ID=CRD42022371498).

### Inclusion criteria

1. English literature;
2. Comparative studies among different resection margin width groups in patients with ICC;
3. Studies provide survival outcomes of patients with ICC who underwent curative surgery.

### Exclusion criteria

1. Abstracts, conference meetings, reviews or letters;
2. Inadequate original date not allowing for further analysis;
3. Studies based on overlapping cohorts derived from the same center.

### Data extraction

Two reviewers assessed all the literature included, and any disagreement was settled by discussion or by another reviewer assessing. The baseline characteristics extracted from each study are summarized in Table 1, including the author, publication year, patient characteristics, interventions, and quality scores (NOS). Odds ratios (ORs) were extracted from Kaplan–Meier curves or extracted directly from the original data.

**Table 1:** Baseline characteristics of involved studies

Study	Center	Study year	Age	Gender (M/F)	pTNM staging (I+II/III+IV)	Tumor differentiation (well moderate/poor)	Size of primary tumor, cm	Lymph node metastases	Mass-forming type	CA19-9 (U/ml)	CEA	Blood transfusion	Adjuvant chemotherapy	Quality score
Bartsch, 2020 [20]	University Medical Center of the Johannes Gutenberg-University Mainz, Germany	2008-2018	64.2 (32.3-84.4)	73/77	60/63	NA	NA	63	NA	NA	NA	NA	NA	8
Cho, 2010 [21]	National Cancer Center, Korea	2001-2007	61.4 (27-82)	41/22	NA	28/32	6.4 (1.1-19)	32	45/18	75.6 (5.0-7590.0)	NA	6	8	8
Farges, 2011 [22]	Multicenter, European	1998-2008	NA	108/104	134/78	NA	7.3	0	212/0	NA	NA	NA	40	7
Liu, 2021 [23]	Multicenter, China	20011-2017	58 (49–64)	287/191	290/88	375/203	6.7	88	154/324	NA	NA	84	97	9
Ma, 2016 [9]	The University of Hong Kong, China	1991-2013	61 (25–79)	58/49	70/37	57/26	6 (1–17)	14	NA	NA	2.4 (0.3–151)	27	NA	8
Shimada, 2007 [24]	National Cancer Center Hospital, Japan	1995-2004	65	32/25	NA	NA	5.2	19	57/0	126	3	NA	NA	7
Tamandl, 2008 [17]	Medical University of Vienna, Austria	1994-2007	63.2 (33.2-85.8)	29/45	NA	NA	NA	23	NA	NA	NA	NA	27	8
Watanabe,2020 [16]	Multicenter, Japan	2000-2017	NA	388/247	231/383	546/113	4.3 (2.8-6.5)	152	401/234	NA	NA	NA	NA	8
Zhang, 2017 [25]	Multicenter, worldwide	1990-2016	59 (51-68)	569/454	776/173	811/117	6.2 (3.6-9.5)	461	817	63.2 (11.2-341.3)	2.4 (1.3-4.6)	354	NA	7
Zheng, 2018 [26]	Peking Union Medical College, China	2007-2016	54.9 (33-76)	45/25	38/32	3/30/31	NA	13	NA	NA	NA	12	NA	7
Zhu, 2021 [27]	Fudan University Shanghai Cancer Center, China	NA	59	66/60	96/30	68/51/7	6.4	33	118/8	67.00 (14.45-335.73)	2.71 (1.73-6.62)	NA	43	8

*NA Not available

### Quality assessment

We used the modified Newcastle–Ottawa Scale (NOS) to assess the quality of these articles [28]. Quality scores >6 were defined as high quality, 4–6 stars as medium quality, and less than 4 stars as low quality.

### Statistical analysis

RevMan 5.3 software was used for statistical analysis. The odds ratio (OR) was applied in dichotomous variable analyses. The pooled OR with its 95% confidence interval (CI) did not overlap with 1, indicating that there was a significant difference. Cochran’s Q-test and the Higgins *I*^2^ statistic were conducted to assess the heterogeneity; significant heterogeneity was defined as a *P* value lower than 0.10 or *I*^2^ greater than 50%. When *I*^2^ <50%, the fixed-effects model was used to estimate the case with homogeneity, and the random-effects model was used for the cases with I^2^ >50% [28]. Any studies that led to high heterogeneity were removed. For sensitivity analysis, we deleted each single study in turn and then analyzed the remaining articles to judge whether the results were significantly affected by a single study [29]. Funnel plots were used for further validation.

## Result

### Study selection and identification

**Fig. 1:**
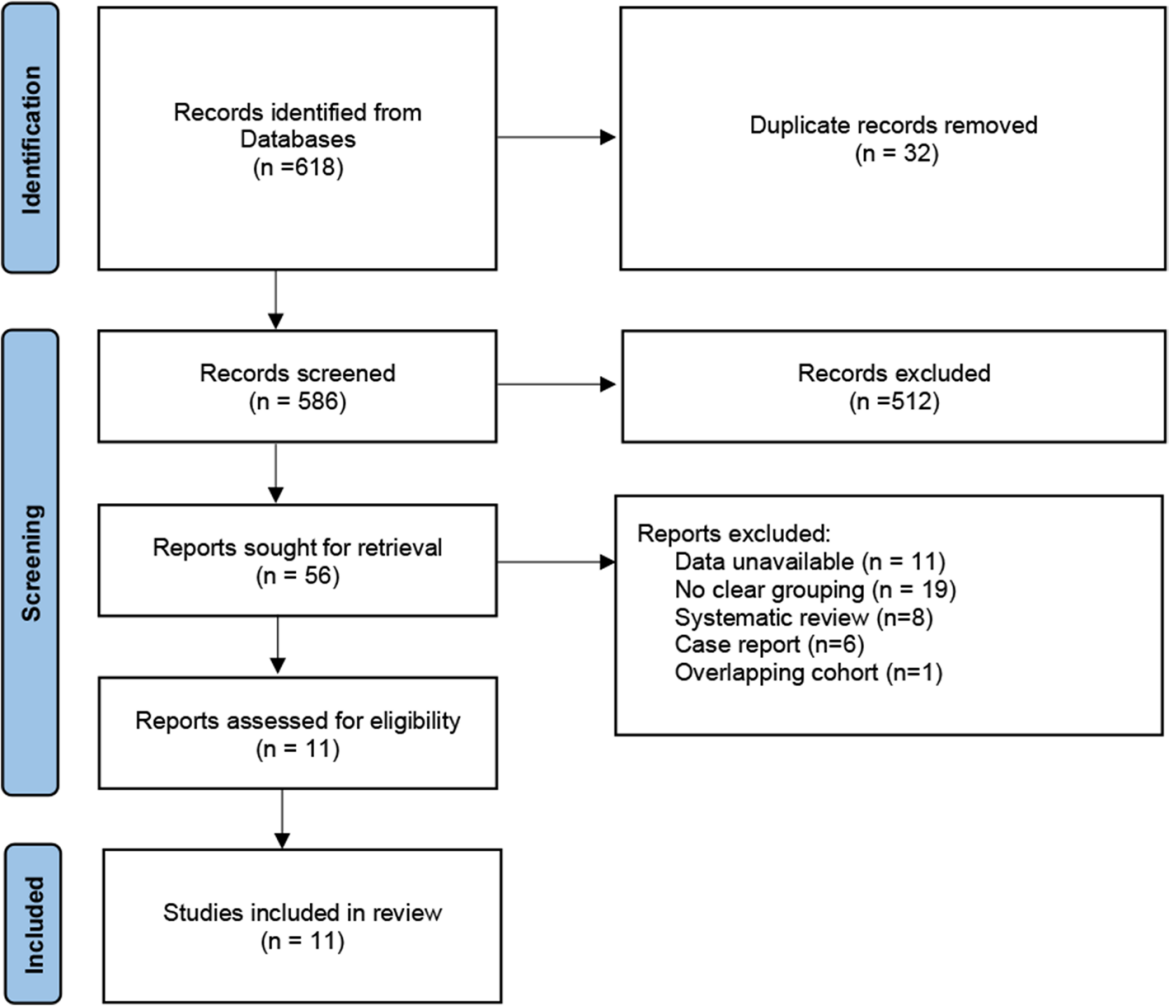
PRISMA flowchart of the study selection

At the outset, 618 relevant articles were discovered and reviewed by two independent researchers, with 32 articles being deleted due to duplication. There were 56 records left after looking through the title and abstract. After applying the inclusion and exclusion criteria, 11 studies were found, none of which were RCTs [9, 16, 17, 20–27]. The results are displayed in Fig. 1.

### Study characteristics

This meta-analysis included 11 studies with a total of 3007 patients, including 1937 patients in the narrow margin group and 1070 patients in the wide margin group. The 11 studies all compared the narrow-margin group to the wide-margin group. The proportion of patients achieving positive margins in the primary operation ranged from 20% to 57% in the 11 studies included. Table 1 shows the characteristics and baseline data from each study, including the author, center, study year, gender, tumor stage, tumor diameter, lymph node metastases, mass-forming type, CA-199, CEA, adjuvant chemotherapy, and quality score. The details of resection in each study are shown in Table Supplementary 1.

### Methodological quality of the included studies

The quality of each included study is shown in Table 1, and the specific evaluation process is listed in Table Supplementary 2. Eleven studies were assessed to be of high quality, none of medium quality, or low quality.

### Overall survival and recurrence-free survival

**Fig. 2:**
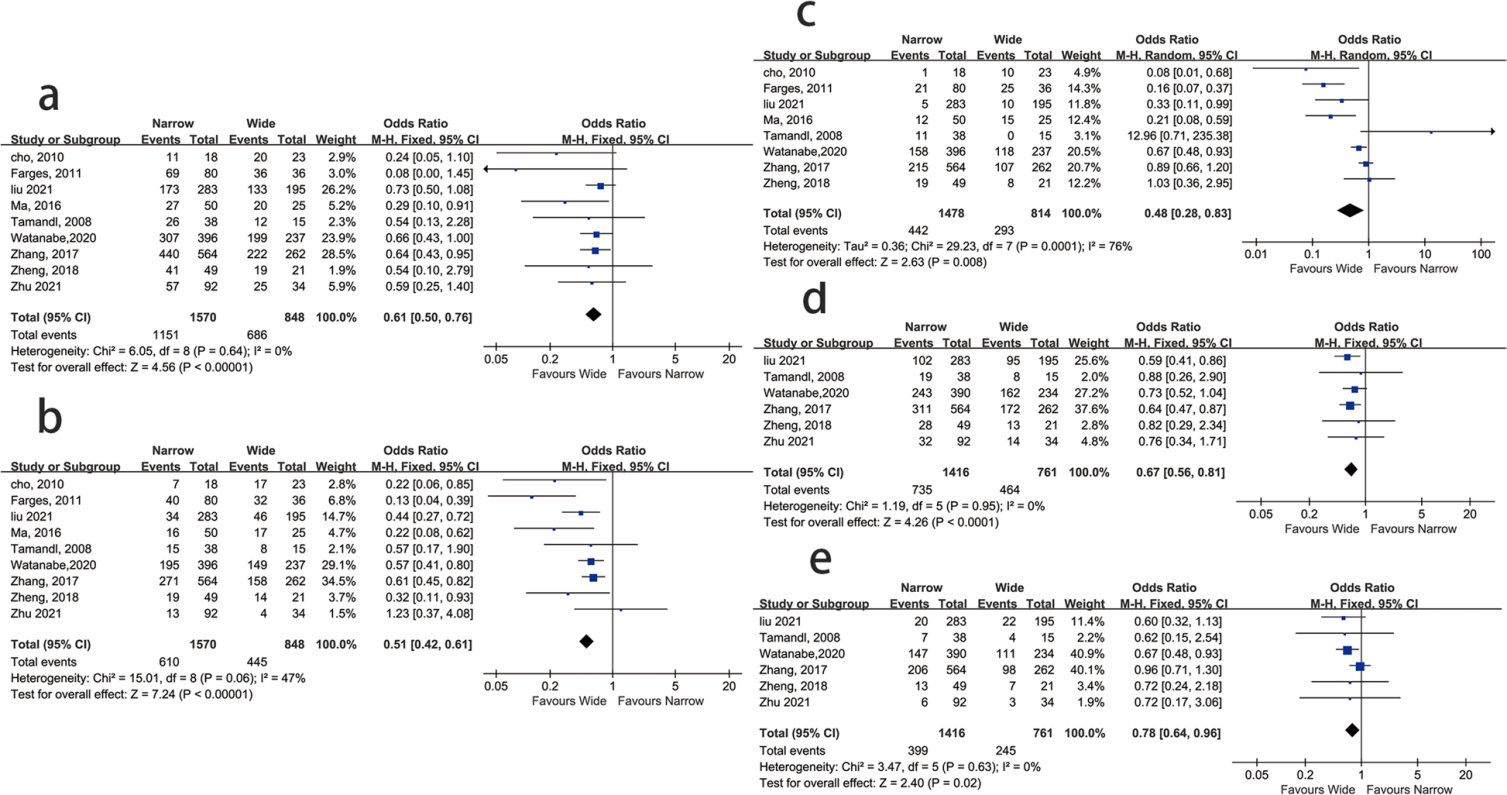
The **a** 1-, **b** 3-, and **c** 5-year overall survival (OS) rates and the **d** 1-, **e** 3-, and **f** 5-year recurrence-free survival (RFS) of the <10-mm margin group vs. the ≥10-mm margin group

In nine studies, the 1-, 3-, and 5-year overall survival (OS) rates were compared between the <10-mm margin group and the ≥10-mm margin group. A wide margin was defined as a resection margin length ≥10 mm, while a narrow margin was defined as a resection margin length <10 mm. Six studies also reported 1-, 3-, and 5-year recurrence-free survival (RFS) rates. The narrow group had a significantly lower 1-, 3- and 5-year OS rate than the wide group (OR = 0.61, 95% CI 0.50–0.76, *P* < 0.01, Fig 2a; OR = 0.51, 95% CI 0.42–0.61, P < 0.01, Fig 2b; OR = 0.48, 95% CI 0.28–0.83, P < 0.01, Fig 2c). Similarly, the 1-, 3-, and 5-year RFS rates showed a worse outcome in the narrow group versus the wide group (OR = 0.67, 95% CI 0.56–0.81, *P* < 0.01, Fig 2d; OR = 0.78, 95% CI 0.64–0.96, *P* = 0.02, Fig 2e; OR = 0.50, 95% CI 0.40–0.63, *P* < 0.001, Fig 2f).

### Location of recurrence

In this meta-analysis, the recurrence rate ranged from 55.7% to 70.84%. Three studies showed recurrence location data, and no significant difference was revealed between the narrow and wide groups (OR = 1.67, 95% CI 0.79–3.53, *P* = 0.18 Figure Supplementary 1a; OR = 1.13, 95% CI 0.43–3.01, *P* = 0.80, Figure Supplementary 1b).

### Subgroup analysis for the different width margin

**Fig. 3:**
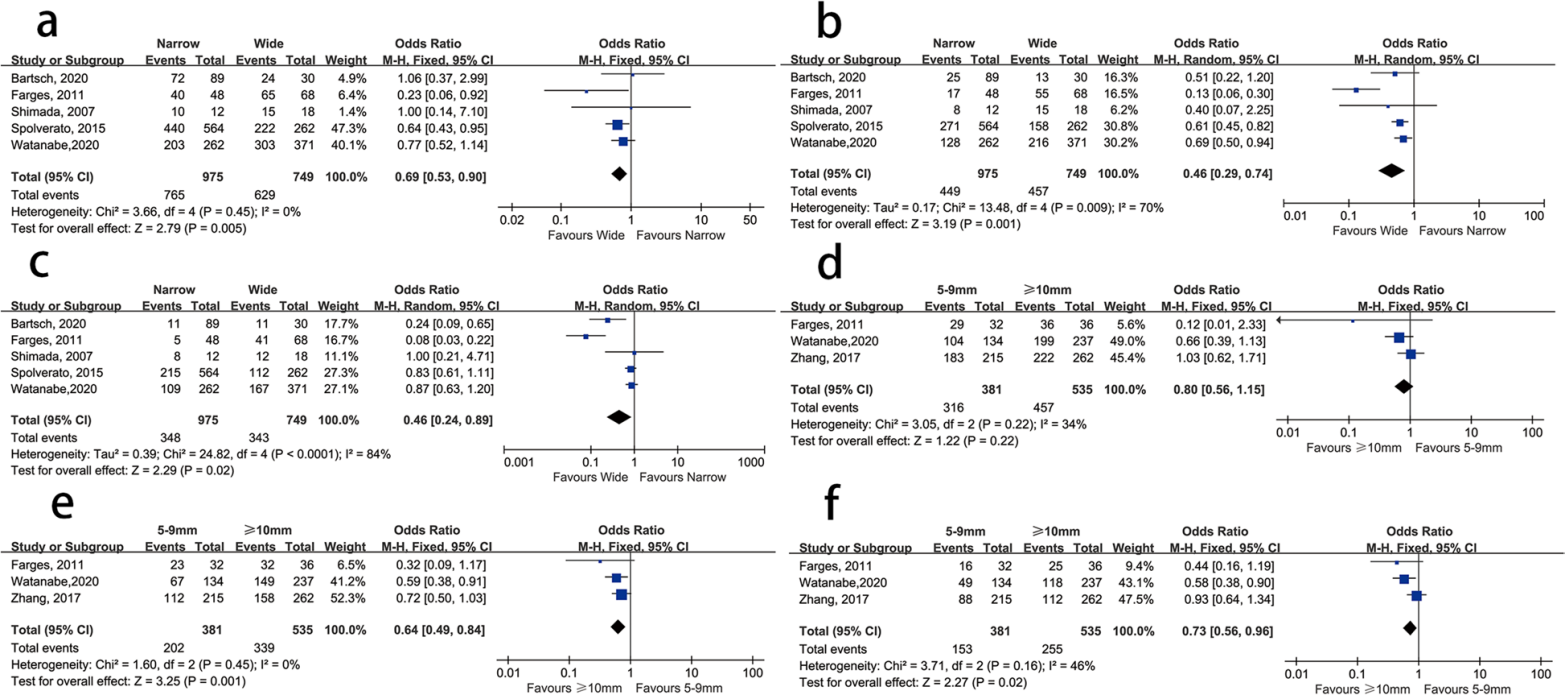
**a–c** 1-, 3-, and 5-year overall survival (OS) rates of the <5-mm margin group vs. the ≥5-mm margin group and **d–f** the 5–9-mm margin group vs. the ≥10-mm margin group

According to the margin width, we divided the narrow group into <5-mm groups and 5–9-mm groups. Compared with the <5-mm group and ≥5-mm group, the wider (5–9-mm group and ≥10-mm group) margin had a significant advantage in the 1-, 3-, and 5-year OS rate (OR = 0.69, 95% CI 0.53–0.90, *P* = 0.005; OR = 0.46, 95% CI 0.29–0.74, *P* < 0.01; OR = 0.46, 95% CI 0.24–0.89, *P* = 0.02; Fig 3). Next, we compared the 1-, 3-, and 5-year OS rates between the 5–9-mm group and the ≥10-mm group, and the results revealed that the survival of the ≥10-mm group was obviously better after the third year after the operation (OR = 0.79, 95% CI 0.47–1.31, *P* = 0.36; OR = 0.64, 95% CI 0.49–0.84, *P* < 0.01; OR = 0.73, 95% CI 0.56–0.96, *P* = 0.02; Fig. 3).

### Subgroup analysis for lymph node status

Two studies revealed an association between lymph node status and the width of the resection margin. In the lymph node-negative subgroup, the 1-, 3-, and 5-year OS rates of the ≥5-mm group (5–9-mm group and ≥10-mm group) were significantly better than those of the <5-mm group (OR = 0.31, 95% CI 0.18–0.53, *P* < 0.01; OR = 0.27, 95% CI 0.07–1.01, *P* = 0.05; OR = 0.40, 95% CI 0.28–0.58, *P* < 0.01, Table Supplementary 3). Similarly, obviously better survival was shown between the ≥10-mm group versus the <10-mm group (OR = 0.44, 95% CI 0.24–0.81, *P* < 0.01; OR = 0.28, 95% CI 0.07–1.06, *P* = 0.06; OR = 0.29, 95% CI 0.10–0.84, *P* = 0.02, Table Supplementary 3). In addition, the comparison made between the <5-mm group and the 5–9-mm group also revealed a significantly better survival in the later group (OR = 1.01, 95% CI 0.41–2.48, *P* = 0.98; OR = 0.58, 95% CI 0.35–0.97, *P* < 0.01; OR = 0.50, 95% CI 0.31–0.80, *P* < 0.01, Table Supplementary 3).

### Complications

Five studies described the complications after ICC resection. In these studies, the postoperative morbidity of ICC was 27%–77.4%, the incidence of minor complications (CDC I–II) was 11.3%–21.6%, and the major complication (CDC III–IV) rate was 13.8%–26%. The 30-day mortality ranged from 4.8% to 8%, and the highest postoperative mortality (CDC V) was 9.5%.

### Prognostic factors

A total of nine studies analyzed prognostic factors through univariate and multivariate analyses. Apart from resection margin width, eight prognostic factors were considered to be risk factors for OS, including lymph node metastasis, vascular invasion, multifocality, tumor size, tumor stage, tumor differentiation satellite nodules, and HBV (hepatitis B virus) infection (Table Supplementary 4). Ten prognostic factors were considered to be independent risk factors for RFS, including tumor size, vascular invasion, lymph node metastasis, multifocality, satellite nodules, tumor stage, tumor differentiation, perineural invasion, bile duct invasion, and level of CEA (Table Supplementary 4).

### Sensitivity analysis and publication bias

In the sensitivity analysis, having removed every single study to re-evaluate the stability of the overall outcomes, no significant difference was detected. Regarding publication bias, Begg’s test and Egger’s test were used for systematic evaluation, and no significant bias was detected (Fig Supplementary 2).

## Discussion

ICC therapy is receiving increasing attention since the incidence rate of ICC has gradually increased over decades. The sole known treatment for curing ICC is curative resection, although the proper margin width is still debatable. This meta-analysis included a total of 3007 patients from eleven studies. The findings revealed that achieving a wider margin by resection was important for improving the prognosis. The findings revealed that a margin width of more than 5 or 10 mm was significant in improving survival. As a result, we compared the difference in overall survival between groups with resection margin widths of 5–10 and ≥10 mm, finding that the latter group had a higher survival rate. As a result, we believe that if we strive for a margin width of more than 10 mm, the prognosis will improve. We should ensure that the resection margin width is larger than 5 mm when the resection margin width cannot be greater than 10 mm. Narrow margins are associated with a worse prognosis due to microvascular invasive lesions, while a broader margin can better eradicate microvascular lesions around the tumor [30]. ICC is thought to have a unique transmission mode [31]. It invades the neighboring liver parenchyma directly through the blood sinus, resulting in the majority of micrometastasis being concentrated in a small area near the tumor. Furthermore, the biological properties of the tumor may play a role. Hepatic parenchymal infiltration is related to MF, periportal vein infiltration is associated with PI, and intraductal diffusion is associated with IG. Patients with IG type have the best prognosis, whereas patients with PI type have the worst prognosis [32–35]. The pathological features of ICC are bile duct invasion, blood vessel invasion, and intrahepatic metastasis [16, 24]. In the most common type of ICC, MF, it is reported that tumor cells exist within only 5 mm of the resection margin because of rare tumor cell proliferation [36]. It should be noted that an excessively long margin did not improve survival. Zhu et al. pointed out that when the resection margin width is greater than 15 mm, there is no significant difference in OS when compared with a resection margin width greater than 10 mm [27]. Furthermore, the narrow margin group’s worse prognosis may be related to the larger, multiple, and later tumor stage compared to the wide margin group, and patients with a single small tumor are more likely to achieve a wide margin, so these factors should be adjusted in future research by creating a comparable group through matching.

ICC has a high propensity for recurrence. According to studies, the postoperative recurrence rate ranges from 55 to 72% [11, 17, 37–39]. According to the results of our current study, it is obvious that a wider margin can extend the RFS of ICC, which is still another reason to pursue a wider resection margin. The recurrence rate in the wide margin and narrow margin groups was equal, according to Tamandl et al. and Watanabe et al. [16, 17]. The studies by Zhu et al., Liu et al. and Zheng et al., on the other hand, found that the width of the margin can greatly reduce the recurrence rate [26, 27, 40]. The data showed that the margin width had no significant effect on the location of recurrence.

After univariate and multivariate analyses, many factors were considered to be related to survival, including multifocality, tumor size, tumor stage, vascular invasion, lymph node metastasis, satellite nodules, CEA, perineural invasion, and bile duct invasion. Although these risk factors are usually comparable between groups in the studies we included, hierarchical analysis based on these risk factors is necessary, which may contribute to future treatment decisions. Studies have pointed out that CA-199 also has an impact on the prognosis of ICC; a lower level of CA-199 may be associated with a better prognosis, but the selection of different cutoff values leads to difficulties in data statistics [23, 27]. In addition, hepatitis B virus infection is also considered to be associated with the prognosis of ICC in some studies, possibly because HBV infection can aggravate cirrhosis and reduce the volume of resectable liver [41–43].

According to certain research, lymph node status is a crucial factor impacting survival in ICC following resection, and lymph node dissection should be performed on a regular basis [18, 41, 44–46]. Inoue et al. proposed that lymph node metastasis showed an unresectable tumor in MF-type ICC [18]. However, few studies have focused on the relationship between lymph node metastasis and incision margin width in ICC. Watanabe et al. demonstrated that in ICC patients with lymph node metastasis, a wider surgical margin did not benefit patients [16]. Farges et al. reached a similar conclusion [22]. He pointed out that a wide margin was associated with a better prognosis only in ICC patients with negative lymph nodes. Therefore, the effect of pursuing a wide margin in patients with positive lymph nodes is questionable, but in patients with negative lymph nodes or unknown lymph node status, wider margins should be guaranteed as far as possible to improve the prognosis of patients. It is expected to provide more evidence of lymph node stratification in the future.

The American Joint Committee on Cancer (AJCC) staging system is the most generally used staging system in the clinic. Tumor stage is one of the most critical factors impacting the prognosis of ICC [47]. Despite the fact that many studies suggest that tumor stage is a predictive factor for ICC, only Liu et al. revealed a link between tumor stage and survival [23]. Only patients in AJCC stage I with a large margin can enhance survival, according to Liu et al., while patients in AJCC stages II and III cannot benefit from a broad margin. This could be due to ICC’s high invasiveness and the condition of the tumor lymph nodes [48].

Many studies have pointed out that a wider resection margin means more complications [16]. To achieve a wider negative margin, more liver resection was performed. Due to the late onset of cirrhosis in ICC patients, more patients can tolerate major hepatectomy [25]. However, a larger resection range cannot improve the survival of ICC patients but also increases the occurrence of complications. In fact, the major hepatectomy group and minor hepatectomy group had similar survival rates [25]. The postoperative morbidity of ICC is as high as 23–78%, the common postoperative complications are biliary fistula, liver abscess, liver failure, cholangitis, intra-abdominal abscess, and so on, and the perioperative mortality is usually less than 10% [18, 32, 37, 49]. In addition, studies have shown that patients in the wide margin group have a higher probability of adverse events, such as intraoperative bleeding and blood transfusion. Therefore, it may be more recommended to ensure that the width of the resection margin is wide enough while pursuing liver parenchyma preservation as much as possible.

Adjuvant therapy is often used as an important method of tumor treatment, but the therapeutic effect of adjuvant therapy on ICC is still unestablished [50–52]. A study has shown that the prognosis of ICC patients in the narrow-margin group can be similar to that in the wide-margin group after radiotherapy [26]. For advanced ICC cancer, gemcitabine combined with cisplatin can significantly improve the survival of patients [53]. However, in resectable ICC, few centers use adjuvant chemotherapy as routine treatments. Some retrospective studies have shown that adjuvant radiotherapy is beneficial for patients with ICC, although some studies disagree. Concurrent chemoradiotherapy has also been reported to improve the prognosis of ICC patients [54]. However, research on the relationship between margin width and adjuvant therapy is still very scarce, and adjuvant therapy may play an important role in the future.

There are some limitations in this meta-analysis. First, the articles included in this meta-analysis were retrospective studies, so recall bias and selection bias were inevitable. Second, because of the low incidence rate of ICC, some studies are small, which reduces the reliability of the results. In addition, due to the lack of relevant data, many subgroup analyses cannot be carried out, such as tumor stage, which may cause the results to be affected by secondary factors. Fourth, due to different surgical methods and instruments, the evaluation of margin width in different centers may not be completely accurate, especially in earlier studies. Finally, although no significant publication bias was observed, it may still exist.

## Conclusion

In conclusion, for resectable ICC, a wide negative margin is helpful to improve the prognosis of patients compared with a narrow negative margin. A resection margin width of over 10 mm is recommended, especially in patients with negative lymph node and early tumor stage. When the resection margin width cannot be greater than 10 mm, we should ensure that the resection margin width is greater than 5 mm. Further high-quality studies will be required to support this conclusion and for analysis.

## Supplementary Information

**Additional file 1: Figure Supplementary 1.** Location of recurrence in the a) ≥10-mm margin group and <10-mm margin group.

**Additional file 2: Figure Supplementary 2.** Funnel diagram of publication bias.

**Additional file 3: Table Supplementary 1.** Surgical procedure of involved studies. **Table Supplementary 2.** Newcastle–Ottawa Scale (NOS) score of involved studies. **Table Supplementary 3.** Subgroup analysis for Lymph node status. **Table Supplementary 4.** Prognostic factors.

## Data Availability

The datasets generated and/or analyzed during the current study are available in the Cochrane Library, PubMed, Embase, MedLine, and Web of Science repository, https://www.cochranelibrary.com/; https://pubmed.ncbi.nlm.nih.gov/; https://www.embase.com/landing?status=grey; http://ovidsp.ovid.com/autologin.html; https://www.webofscience.com/wos/alldb/basic-search.

## Abbreviations

ICC: Intrahepatic cholangiocarcinoma
MF: Mass-forming
IG: Intraductal growth
PI: Periductal infiltrative
MST: Median survival time
ORs: Odds ratios
NOS: Newcastle–Ottawa Scale
CI: Confidence
OS: Overall survival
RFS: Recurrence-free survival
HBV: Hepatitis B virus
