# Supplementary figures and images for: The influence of resection margin width in patients with intrahepatic cholangiocarcinoma: a meta-analysis
Source: World J Surg Oncol. 2023 Jan 20;21:16. doi: 10.1186/s12957-023-02901-5 (PMC9854153; doi:10.1186/s12957-023-02901-5)

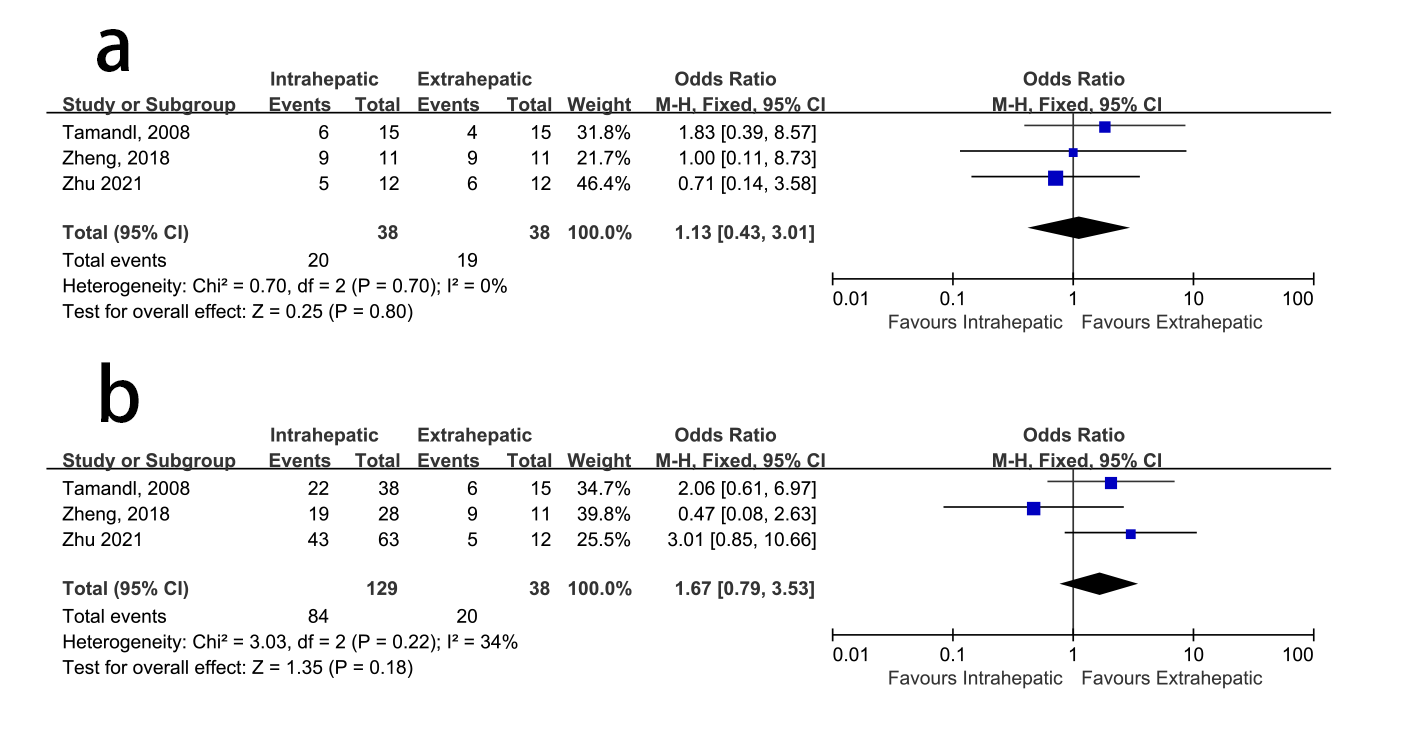

Supplement: Supplementary file 1 — Additional file 1: Figure Supplementary 1. Location of recurrence in the a) ≥10-mm margin group and <10-mm margin group. [file 12957_2023_2901_MOESM1_ESM.tif]

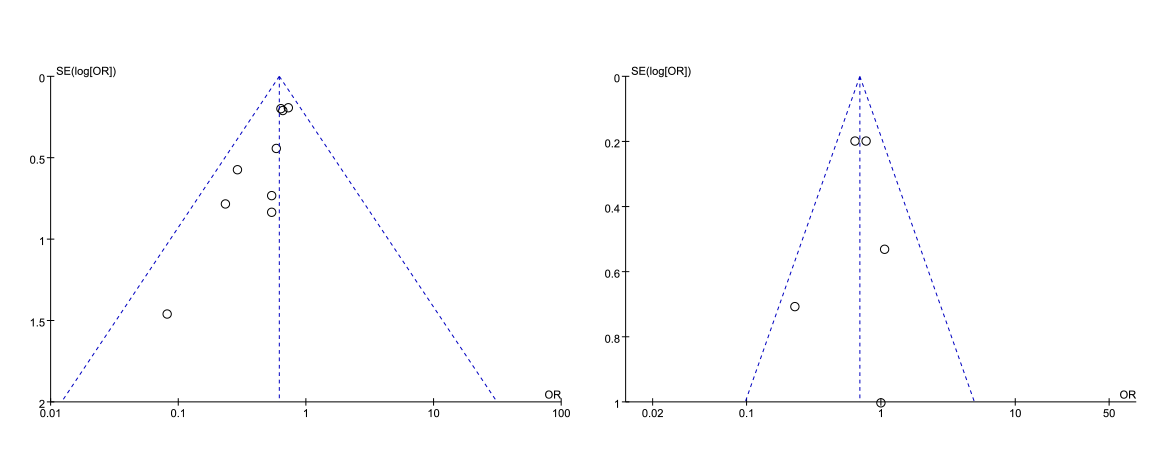

Supplement: Supplementary file 2 — Additional file 2: Figure Supplementary 2. Funnel diagram of publication bias. [file 12957_2023_2901_MOESM2_ESM.tif]
