# Supplementary material for: The influence of resection margin width in patients with intrahepatic cholangiocarcinoma: a meta-analysis
Source: World J Surg Oncol. 2023 Jan 20;21:16. doi: 10.1186/s12957-023-02901-5 (PMC9854153; doi:10.1186/s12957-023-02901-5)
Supplement: Supplementary file 3 — Additional file 3: Table Supplementary 1. Surgical procedure of involved studies. Table Supplementary 2. Newcastle–Ottawa Scale (NOS) score of involved studies. Table Supplementary 3. Subgroup analysis for Lymph node status. Table Supplementary 4. Prognostic factors. [file 12957_2023_2901_MOESM3_ESM.docx]

Table Supplementary 1. Surgical procedure of involved studies

| Major hepatectomy | | | | | | | | | Minor hepatectomy | | | | |
| --- | --- | --- | --- | --- | --- | --- | --- | --- | --- | --- | --- | --- | --- |
|  | Total | Right hepatectomy | Extended right hepatectomy | Left hepatectomy | Extended left hepatectomy | Right trisectionectomy | Left trisectionectomy | Others | Total | Left lateral sectionectomy | Segmentectomy | Subsegmentectomy | Others |
| Bartsch, 2020 | 101 | 25 | NA | 19 | NA | 26 | 22 | 9 | 25 | NA | 34 | NA | NA |
| cho, 2010 | 49 | 21 | 2 | 8 | 12 | 3 | 1 | 1 | 14 | 2 | 5 | NA | NA |
| Farges, 2011 | 182 | | | | | | | | 40 | | | | |
| liu, 2021 | 326 | | | | | | | | 152 | | | | |
| Ma, 2016 | 73 | 13 | 5 | 11 | 7 | 16 | 2 | NA | 52 | 10 | 16 | 6 | 21 |
| Tamandl, 2008 | 41 | NA | 16 | 14 | NA | 8 | 3 | NA | 33 | 6 | 6 | NA | 21 |
| Zhang，2017 | 608 | 161 | 128 | 202 | 99 | 18 | | NA | 415 | NA | 254 | NA | 161 |
| Watanabe,2020 | NA | | | | | | | | | | | | |
| Zheng, 2018 | NA | | | | | | | | | | | | |
| Zhu, 2021 | NA | | | | | | | | | | | | |

*NA: not available

Table Supplementary 2. Newcastle–Ottawa Scale (NOS) score of involved studies

|  | SELECTION | | | | COMPARABILITY | EXPOSURE | | |  |
| --- | --- | --- | --- | --- | --- | --- | --- | --- | --- |
| Study | Representativeness of the exposed cohort | Selection of the non exposed cohort | Ascertainment of exposure | Demonstration that outcome of interest was not present at start of study | Comparability of cohorts on the basis of the design or analysis | Assessment of outcome | Was follow-up long enough for outcomes to occur | Adequacy of follow up of cohorts | Quality score |
| Bartsch, 2020 | ★ | ★ | ★ | ★ | ★ | ★ | ★ | ★ | 8 |
| cho, 2010 | ★ | ★ | ★ | ★ | ★ | ★ | ★ | ★ | 8 |
| Farges, 2011 | ☆ | ★ | ★ | ★ | ★ | ★ | ★ | ★ | 7 |
| liu, 2021 | ★ | ★ | ★ | ★ | ★★ | ★ | ★ | ★ | 9 |
| Ma, 2016 | ★ | ★ | ★ | ★ | ★ | ★ | ★ | ★ | 8 |
| Shimada, 2007 | ☆ | ★ | ★ | ★ | ★ | ★ | ★ | ★ | 7 |
| Tamandl, 2008 | ★ | ★ | ★ | ★ | ★ | ★ | ★ | ★ | 8 |
| Watanabe,2020 | ★ | ★ | ★ | ★ | ★ | ★ | ★ | ★ | 8 |
| Zhang, 2017 | ★ | ★ | ★ | ★ | ★ | ★ | ★ | ☆ | 7 |
| Zheng, 2018 | ☆ | ★ | ★ | ★ | ★ | ★ | ★ | ★ | 7 |
| Zhu, 2021 | ★ | ★ | ★ | ★ | ★ | ★ | ★ | ★ | 8 |

Table Supplementary 3. Subgroup analysis for Lymph node status

|  |  | OR | 95%CI | P value |
| --- | --- | --- | --- | --- |
| ≥5mm versus <5mm | 1- year OS rate | 0.31 | 0.18-0.53 | <0.01 |
|  | 3- year OS rate | 0.27 | 0.07-1.01 | 0.05 |
|  | 5- year OS rate | 0.40 | 0.28-0.58 | <0.01 |
| 5-9mm versus ≥10mm | 1- year OS rate | 1.01 | 0.41-2.48 | 0.98 |
|  | 3- year OS rate | 0.58 | 0.35-0.97 | <0.01 |
|  | 5- year OS rate | 0.5 | 0.31-0.80 | <0.01 |
| ≥10mm versus <10mm | 1- year OS rate | 0.44 | 0.24-0.81 | <0.01 |
|  | 3- year OS rate | 0.28 | 0.07-1.06 | 0.06 |
|  | 5- year OS rate | 0.29 | 0.10-0.84 | 0.02 |

Table Supplementary 4. Prognostic factors

|  | Factor | RR | 95%CI | Studies involved |
| --- | --- | --- | --- | --- |
| Overall survival | Lymph node metastasis | 2.35 | 1.54-3.60 | 7 |
|  | Vascular invasion | 1.53 | 1.29-1.82 | 5 |
|  | Tumor size> 5cm | 1.53 | 1.30-1.80 | 4 |
|  | Tumor differentiation | 1.51 | 1.27-1.80 | 4 |
|  | Satellite nodules | 1.36 | 1.08-1.73 | 4 |
|  | Multifocality | 1.88 | 1.58-2.23 | 4 |
|  | Tumor stage | 2.84 | 2.26-3.57 | 3 |
|  | HBV infection | 1.15 | 1.11-1.20 | 2 |
| Recurrence-free survival | Lymph node metastasis | 1.57 | 1.02-2.43 | 6 |
|  | Vascular invasion | 1.82 | 1.53-2.18 | 5 |
|  | Tumor size> 5cm | 1.61 | 1.36-1.90 | 5 |
|  | Multifocality | 1.55 | 1.25-1.92 | 4 |
|  | Satellite nodules | 1.16 | 0.92-1.47 | 4 |
|  | Tumor stage | 2.09 | 1.36-3.21 | 3 |
|  | Tumor differentiation | 1.55 | 1.28-1.88 | 3 |
|  | Perineural invasion | 1.47 | 1.15-1.87 | 3 |
|  | CEA>5ng/ml | 2.24 | 1.43-3.51 | 3 |
|  | Bile duct invation | 1.41 | 1.20-1.65 | 2 |
